# Concentrations of ^137^Cs in forest mushrooms and soil in southern Poland

**DOI:** 10.2478/jvetres-2026-0002

**Published:** 2026-01-13

**Authors:** Magdalena Gembal, Paweł Czerski, Małgorzata Warenik-Bany

**Affiliations:** Department of Chemical Research of Food and Feed, National Veterinary Research Institute, 24-100 Pulawy, Poland

**Keywords:** ^137^Cs, Chernobyl, legacy radionuclide contamination, mushrooms, soil-to-fungal transfer

## Abstract

**Introduction:**

Since 1986, Poland has been constantly contaminated with radioactive ^137^Cs, and before that, it experienced fallout like most of the world since nuclear bomb tests. As a result of radioactive fallout, vegetation and fungi acquired contamination. The distribution and migration of ^137^Cs between elements of the environment affect the uptake of the radionuclide by animals and its movement up the food chain. This research was conducted to measure the concentrations of ^137^Cs in soil and mushrooms and to understand radionuclide transfer.

**Material and Methods:**

The research material consisted of mushroom and soil samples obtained from a forest in southern Poland. A total of 30 fungi and 20 soil samples were tested using gamma radiation spectrometry.

**Results:**

The highest concentration in a whole-mushroom sample was 1,650 ± 20.40 Bq/kg, in a stipe was 2,192 ± 264.1 Bq/kg and in a cap was 1,323 ± 159.5 Bq/kg. The highest concentration in a soil sample taken from a depth of 0–10 cm was 105.7 ± 13.15 Bq/kg and in a sample from a depth of 10–20 cm was 39.48 ± 5.310 Bq/kg.

**Conclusion:**

The obtained results indicate that the transfer of ^137^Cs occurs from the soil towards the mushroom fruiting bodies, with the highest concentrations recorded in the stipes. Additionally, the cross-section of the transport profile shows the distribution of the tested radionuclide in individual elements of the environment and thus the degree of their radioactive contamination.

## Introduction

Fungi are organisms that differ from plants not only in appearance, but also in chemical composition, manner of growth and how they sustain themselves. Some scientists have even considered them to be a third completely separate kingdom from plants and animals (4). Even though mushrooms grow almost everywhere, they favour soil and wood substrates (3) and are most often found in forests, where the substances that they need to live are abundant. The fruiting bodies of some mushrooms bioaccumulate minerals and other nutrients, and are therefore valuable food sources. Man has been eating mushrooms for a very long time. Mushrooms are also food for many free-living animals, especially wild boars, red deer and roe deer. The strong propensity of some mushrooms regularly eaten by humans and animals to accumulate radioisotopes of caesium has been described in the literature. Examples of such mushrooms in Poland are the bay bolete (*Xerocomus badius*) and deer truffle (*Elaphomyces granulatus*), which may be sources of radionuclides. As Mietelski *et al*. (25) pointed out, the accumulation of ^137^Cs in mushroom fruiting bodies was discovered almost 45 years ago. Therefore, food chain penetration by radionuclide contamination is a persistent risk.

Radiocaesium appeared in the environment as a result of global fallout from atmospheric nuclear tests (15). Since the time of testing, much data has been collected (6, 10, 20, 26). The main source of radioactive contamination in Poland and neighbouring countries was the accident at the Chernobyl nuclear power plant in 1986. As a result of this accident, a radioactive cloud spread over Eastern Europe, causing very uneven contamination. One of the most important radionuclides that has penetrated the ecosystem, and therefore created a serious threat to the environment as well as animal and human health, is ^137^Cs, and this was one contaminant released by the Chernobyl explosion. In areas where there was rainfall and a high concentration of radioactive clouds, there was greater contamination. Through wet deposition in the first days after the accident, ^137^Cs was carried from the leaves in rainwater to the surface of the soil litter (9, 30). Studies show that radioactive ^137^Cs enters the soil–plant–herbivore–carnivore chain in this way (7, 11, 31, 32). The vast majority of ^137^Cs was seen to accumulate in the top layer of soil, which is rich in organic and mineral compounds (31). It has been suggested that the transfer of radiocaesium from soil by fungi and plants to animals is much higher in forest ecosystems than in agricultural environments, and the decline in soil caesium concentration is very slow (19, 21, 22, 34). Forest soils are characterised by low potassium, low pH and increased organic matter (Cs is less bound in organic matter compared to clay or silt), implying that its bioavailability is higher in such areas. A dependence of the accumulation of ^137^Cs on the mushroom species was noticed. Some authors explained this feature by the chemical properties of compounds found in fruiting bodies, such as the brown pigment that was discovered and characterised in *Xerocomus badius* (33). Species that strongly accumulate ^137^Cs besides *Xerocomus badius* include *Xerocomus chrysenteron, Suillus variegatus, Rozites caperata* and *Hydnum repandum*, which all grow in areas affected by Chernobyl fallout, and the last two of which are in demand as food. The concentration of the activity of ^137^Cs was reported to be influenced by several environmental factors, such as the degree of contamination with atmospheric precipitation of the soil from which the mycelium draws nutrients, soil moisture content and the time since the nuclear accident (20). Authors indicated the relationship between the properties of radiocaesium accumulation and the depth of mycelium location as the most important factor (13, 14, 24, 27, 29). Accumulation patterns in mushrooms can be compared through transfer factors or aggregation coefficients, defined as the ratio of the activity concentration in the fruiting body to the concentration in the top 10 cm of soil or to the cumulative deposition. These parameters were used to characterise the radiocaesium accumulation properties of fruiting bodies as time-dependent (23).

In Europe, where wild mushrooms are commonly eaten as a delicacy, some species were found to have been significantly contaminated by radioactive fallout from the Chernobyl accident in 1986. Consumption of wild mushrooms contributed to the intake of an effective dose (0.2 mSv) in individuals consuming approximately 10 kg (fresh weight) of the highly contaminated species per year.

The concentration of radioactive isotopes in food products should fall under the values specified in the Regulation of the Council of Ministers in Poland of April 27, 2004 (12), which states that the maximum permissible levels in food products is 1,250 Bq/kg. This level refers to isotopes with a half-life longer than 10 days and mainly concerns ^134^Cs and ^137^Cs. Additionally, in accordance with European law, the activity of radioactive isotopes in foodstuffs and food products should be no more than the values specified in the Commission Implementing Regulation (EU) of 17 January 2024 (8). This states that the concentration of the ^137^Cs isotope in commercial food products cannot exceed 600 Bq/kg in all foodstuffs and products except milk and dairy products, where the concentration of this isotope cannot exceed 370 Bq/kg.

The aims of the study were to determine the content of ^137^Cs in samples of edible and inedible mushrooms randomly collected in a forest region of southern Poland, and also to estimate the degree of exposure of consumers of edible mushrooms to the presence of harmful ionising radiation in them. In order to explain the presence of radioactive radioisotopes in mushrooms, it was necessary to refer to the still-present radioactive contamination of the environment with ^137^Cs, using the example of tested soil samples. Additionally, testing soil samples made it possible to determine the ^137^Cs transport profile between the soil system and the fruiting bodies of fungi. In this way, information was obtained about the current state of radioactive contamination of the environment in the indicated forest area in Poland.

## Material and Methods

### Material for research

**Fig. 1.**
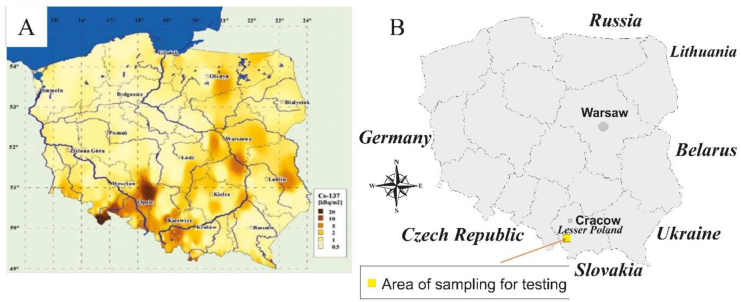
A – The distribution of radioactive fallout in Poland (Isajenko *et al*., 2011); B – sampling site for soil and mushrooms

The research material consisted of mushroom samples and soil samples obtained from forest areas in southern Poland (49°39′41″N and 19°33′38″E) in 2023. The sampling site covered an area of approximately 10 km^2^. Samples were taken randomly and came from different locations in the designated area. Obtaining material for research from wild areas free from human interference allowed us to characterise the natural flow of radionuclides in the environment. Figure 1 shows two maps of Poland. The map on the left (Fig. 1A) is reproduced from the 2011 edition of the Radiation Atlas of Poland (18). It presents the distribution of ^137^Cs concentration in the surface layer (0–10 cm) of soil in Poland and shows that Poland is contaminated with this radionuclide in an uneven manner. The highest concentration of ^137^Cs was observed in south-eastern Poland, in the eastern part of the Sudeten foothills and the Silesian Lowland, around Opole and Kraków. The illustration on the right (Fig. 1B) shows the sampling site, Zawoja. It is an area located in the Malopolskie Voivodeship (near Kraków), which is non-agricultural and sustained by tourism and is situated near the Beskid Zywiecki and Beskid Makowski mountain ranges. The lower mountain forest selected as a sampling area is characterised by stands of spruce and fir with a mixture of beech and sycamore. The soils in these areas are rankers and podzolic types characterised by a rather poor silicate substrate of raw debris.

A total of 30 mushroom samples (10 edible and 20 inedible) and 20 soil samples were examined. Soil and fungal samples were taken simultaneously from a randomly selected site in autumn 2023. Various young-to-mature specimens of ground-growing species of edible mushrooms (*Boletus edulis, Boletus reticulatus, Suillus luteus, Suillus bovinus* (L.) Roussel, *Chroogomphus rutilus, Cantharellus cibarius, Cantharellus pallens* and *Craterellus cornucopioides*) and inedible ones (*Amanita muscaria, Neoboletus luridiformis, Russula sardonia* and *Russula mairei*) were collected.

**Fig. 2.**
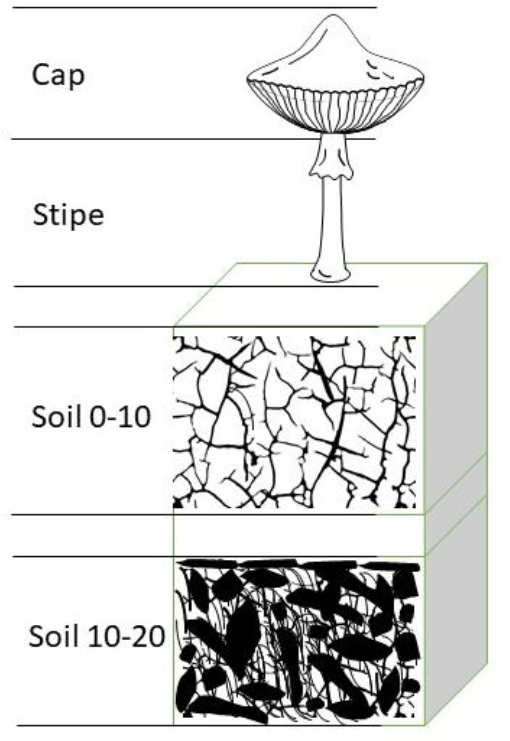
Environmental elements of ^137^Cs transport

The two main elements of the mushroom fruiting bodies (stipe and cap, eligible by being in good condition) were analysed (Fig. 2). One mushroom constituted one sample. Dry weight was not determined; samples weighing approximately 10 g were prepared. Fresh fruiting bodies were cleaned of visible plant and soil residues using a knife and a sterile plastic brush. The lower part of it was cut off from the cap and two separate sub-samples were prepared. Crushed and homogenised samples were transferred to jars. Soil samples were collected at two depths – from 0 to 10 cm and from 10 to 20 cm (Fig. 2). The soil samples were not dried but were sieved and then homogenised. Samples weighing approximately 20 g were prepared. Straight-sided unlined polypropylene jars with white closures and 58 mm cap size with 400 thread were used for analysis. The measuring containers were appropriately calibrated using a specialised calibration source (ISOXSRCE, Eu-155 and Na-22; Mirion Technologies, Atlanta, GA, USA) with certified activity on 20/05/2020 of 0.07 MBq. This allowed determining the proper geometry for measurements of small sample volumes. The analytical tool prepared in this way helped to solve all technical problems and overcome the limitations of the detector using only classical calibration. After measuring the cap and stipe, these sub-samples were combined to measure the entire mushroom.

### Instrumental analysis

**Fig. 3.**
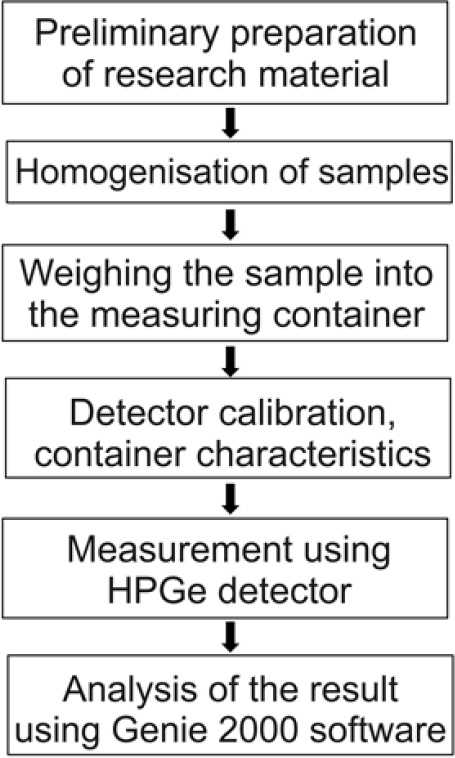
Scheme of performing the analysis

A method accredited by the Polish Centre for Accreditation was used to determine the radioactive concentration of ^137^Cs (28). Gamma radiation spectrometry was conducted with a high-purity P-type germanium semiconductor coaxial detector with a relative efficiency of 25%–35% and an energy tolerance of 1.000 keV full width at half maximum. The measurement time was 72, 000 sec (20 h). The collected gamma-ray spectra were analysed using Genie 2000 software (Mirion Technologies) (Fig. 3). The laboratory has participated in numerous International Atomic Energy Agency proficiency tests using this method and has always passed.

### Statistical analysis

The obtained results were collected and processed to facilitate the reconstitution of the ^137^Cs transport profiles. The average concentration, standard deviation and median were calculated using Excel (Microsoft; Redmond, WA, USA). Additionally, the minimum and maximum measured concentrations were summarised. Statistica v.13.1 was used for statistical evaluation (Dell, Round Rock, TX, USA). The results were evaluated using statistical tests, initially with the Kolmogorov–Smirnov test to check the distribution of the data. The result indicated a non-normal distribution; therefore, nonparametric tests were used in further analysis. The nonparametric Mann–Whitney U Test was applied to compare two independent groups with the dependent variable expressed on an ordinal scale. For samples of small or medium size, the STATISTICA program calculates the exact probability associated with the distribution of the U statistic. The Mann-Whitney test statistic "U" reflects the difference between two rank sums. The results of the Mann-Whitney U test are reported as U and p. If p < 0.05, the result obtained is statistically significant, which suggests that there are differences between the compared groups. Spearman’s rank correlation was also used as a nonparametric measure of monotonic statistical dependence between random variables. The correlation results are reported as p. If p < 0.05 the result obtained is statistically significant, which suggests that there are differences between the compared groups. To assess the transmission direction of ^137^Cs, the transfer factor (TF) commonly used in radioactive studies was determined.

### Determination of the transfer factor

The TF, known as the soil-to-plant concentration ratio, is a key parameter for estimating radionuclide concentration in plants and for use in dose assessment (5). Transfer of ^137^Cs between the individual environmental elements tested can also be determined from the results of the tests and the TF. For the purposes of the experiment, it was calculated using the formula below and expressed in Bq/kg of fresh weight *fw* for soil 0–10 cm, soil 10–20 cm, stipes and caps:

$$\text{TF}=\frac{\;\text{Concentration in plant }(\text{mushrooms})\;(\text{Bq}/\text{kg}\;fw)}{\;\text{Concentration in soil}\;(0-10\;\text{cm},10-20\;\text{cm})(\text{Bq}/\text{kg }fw)}$$

## Results

### Measurement of ^137^Cs concentration

Table 1 shows the concentrations of ^137^Cs along with the uncertainty for the 30 whole-mushroom samples. Concentrations ranged from single-figure amounts to over 2,000 Bq/kg. The average concentration value in the whole-mushroom samples was 359.0 Bq/kg, and the highest concentration in these samples was 1,650 ± 20.40 Bq/kg. The mass of material was insufficient for the cap and stipe to be examined separately in 13 samples. The average concentration in the stipe was 565.2 Bq/kg and in the cap was 322.7 Bq/kg. The highest concentration recorded in the mushroom stipe was 2,192 ± 264.1 Bq/kg and in the mushroom cap was 1,323 ± 159.5 Bq/kg.

**Table 1.** Concentrations of ^137^Cs for the whole mushroom, cap, stipe and two layers of soil

Number	Whole mushrooms	Caps	Stipes	Soil 0–10 cm	Soil 10–20 cm
	Result ± uncertainty (Bq/kg)				
1	12.90 ± 2.100	11.30 ± 4.780	14.70 ± 2.290	57.31 ± 7.601	26.68 ± 3.698
2	30.90 ± 4.250	19.40 ± 2.990	46.60 ± 6.390	59.78 ± 7.767	39.48 ± 5.310
3	3.830 ± 1.110	5.660 ± 2.480	3.480 ± 1.340	53.84 ± 7.159	30.16 ± 4.159
4	98.10 ± 12.20	56.40 ± 8.720	130.3 ± 16.30	60.79 ± 7.968	27.24 ± 3.861
5	875.0 ± 105.5	752.8 ± 91.10	1,248 ± 150.7	75.41 ± 9.537	10.61 ± 1.899
6	802.2 ± 96.90	546.5 ± 66.30	1,246 ± 150.5	99.89 ± 11.38	18.75 ± 2.833
7	1,345 ± 162.0	1,139 ± 137.4	1,986 ± 239.5	105.7 ± 13.15	17.34 ± 2.842
8	90.40 ± 11.60	58.30 ± 9.460	123.3 ± 16.00	54.30 ± 7.173	3.821 ± 1.191
9	26.40 ± 3.990	-	-	55.80 ± 7.177	6.156 ± 1.317
10	10.30 ± 1.870	9.660 ± 2.400	14.00 ± 2.500	55.28 ± 7.113	4.008 ± 0.604
11	1,170 ± 15.10	-	-	-	-
12	97.00 ± 12.10	52.50 ± 7.130	127.5 ± 15.80	-	-
13	19.70 ± 2.940	12.10 ± 2.120	26.60 ± 3.800	-	-
14	365.8 ± 44.40	208.1 ± 26.00	546.9 ± 66.50	-	-
15	165.7 ± 20.50	127.2 ± 16.60	209.7 ± 26.00	-	-
16	13.50 ± 2.260	11.70 ± 2.370	12.50 ± 2.220	-	-
17	93.60 ± 11.70	81.50 ± 10.60	104.0 ± 13.00	-	-
18	759.2 ± 91.80	-	-	-	-
19	50.30 ± 6.830	-	-	-	-
20	26.70 ± 3.990	-	-	-	-
21	17.10 ± 3.700	-	-	-	-
22	679.4 ± 82.30	-	-	-	-
23	1,650 ± 20.40	-	-	-	-
24	1,556 ± 187.3	1,323 ± 159.5	2,192 ± 264.1	-	-
25	169.0 ± 21.10	-	-	-	-
26	38.20 ± 5.740	-	-	-	-
27	130.0 ± 16.50	-	-	-	-
28	1,208 ± 145.5	1,071 ± 129.2	1,577 ± 190.2	-	-
29	204.3 ± 25.10	-	-	-	-
30	108.3 ± 13.50	-	-	-	-
Average	359.0	322.7	565.2	66.91	18.42
SD	553.1	458.7	763.3	18.01	12.30
Median	147.9	58.30	127.5	58.55	18.05
Min ± uncertainty	3.830 ± 1.110	5.660 ± 2.120	3.480 ± 1.340	54.00 ± 7.110	3.821 ± 0.604
Min ± uncertainty	1,650 ± 20.40	1,323 ± 159.5	2,192 ± 264.1	105.7 ± 13.15	39.48 ± 5.310

### Measurement of ^137^Cs concentration in samples of soil layers

The concentrations of ^137^Cs in the soil samples ranged from single-figure amounts to 100 Bq/kg. Table 1 shows detailed data for the 10 soil samples taken at a depth of 0–10 cm and the 10 taken at a depth of 10–20 cm. The mean concentrations were 66.91 Bq/kg and 18.42 Bq/kg, respectively. The highest concentration in a shallower sample was 105.7 ± 13.15 Bq/kg and in a deeper sample was 39.48 ± 5.310 Bq/kg.

### Transport profile of ^137^Cs in the structure and substrate

**Fig. 4.**
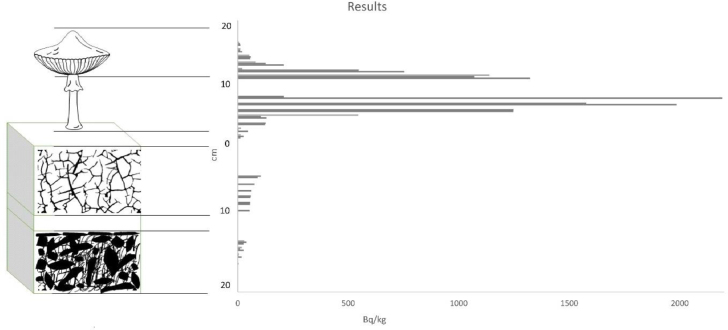
^137^Cs concentrations in individual layers

A graph (Fig. 4) shows the concentrations of ^137^Cs in the 10–20 cm soil layer, the 0–10 cm soil layer and the stipes and caps of the mushrooms. The concentration distribution clearly shows that ^137^Cs was accumulated in the top layer of soil to a greater extent than in the deeper layer of soil. The concentration of ^137^Cs in the mushrooms was also not uniform and was notably higher in the stipes than in the caps.

### Statistical analysis

The results obtained from caps and stipes were compared. The Mann–Whitney U Test gave a value of U of 195.0000 and a p-value of 0.275064. The cap ^137^Cs concentrations did not differ statistically significantly from the stipe concentrations. The linear regression model, based on the Spearman test, did not show any correlation between the data for soil at a depth of 10–20 cm and those for the soil at a depth of 0–10 cm (p-value = 0.651477). It also did not show any correlation between the data for soil at a depth of 0–10 cm and those for the stipe (p-value = 0.883333). However, for the stipe–cap relationship, a correlation was shown between the data (p-value = 0.000000).

### Transfer factor

The collected results allowed the quantitative determination of the transfer of ^137^Cs between the tested environmental elements (Table 2). The transfer factor was determined for 10 samples of mushrooms and soil. For the transfer of ^137^Cs from soil at a depth of 10–20 cm to soil at a depth of 0–10 cm, the average TF was 6.702. The average TF calculated for the transfer between the 0–10 cm soil system and the stipe was 5.707. The factor for the transfer from the stipe to the cap was 0.643. Additionally, an attempt was made to determine the TF for the transfer of ^137^Cs from the substrate (soil) to the entire mushroom fruiting body (stipe and cap).

**Table 2.** Transfer factor determined for individual environmental matrices

Number	Transfer factor		
	Soil 10–20 cm → Soil 0–10 cm	Soil 0–10 cm → Stipes	Stipes → Caps
1	2.146	0.256	0.768
2	1.514	0.779	0.416
3	1.785	0.064	1.626
4	2.231	2.143	0.432
5	7.107	16.54	0.603
6	6.095	13.70	0.438
7	14.21	18.78	0.573
8	9.069	2.270	0.472
9	9.069	2.282	0.411
10	13.79	0.253	0.690
Average	6.702	5.707	0.643
SD	4.833	7.483	0.367

## Discussion

Since the test explosions of nuclear weapons in the atmosphere in the 1960s, various species of mushrooms have often been tested for the accumulation of radiocaesium, and the edible *Xerocomus badius* is a frequent test subject (25). In many studies, it and other fungi are found to accumulate ^137^Cs. Despite 39 years having passed since the Chernobyl accident, radiocaesium contamination of forest areas is still observed, which confirms the accumulation of caesium by various species of forest fungi.

For the tested mushrooms from the forest in southern Poland, the average measured value of the ^137^Cs concentration was 359.0 Bq/kg. The highest concentration in whole fungi samples was 1,650 ± 20.40 Bq/kg. Comparisons of these concentrations with those previously noted in forest areas show strong contrasts: samples of *Xerocomus badius* collected in 1991 in the Opole region, which is 162 km to the northwest of the sampling site, ranged in their averages from 0.4 to 18.2 kBq/kg and reached a maximum of 157 kBq/kg (25). In studies conducted from 1997 to 1998 (36) in the same region, it was found that the concentrations of ^137^Cs varied from 2.6 kBq/kg to 64.8 kBq/kg. Overall, a decrease in concentration over time was observed (more rapidly than the decrease due to the half-life alone), as activity in the range of 100 kBq/kg was never detected again after 1991 (23). Much lower results can be found in countries far from Chernobyl, such as Spain (1). Based on literature data, it can be concluded that among mushrooms collected in one place, their ability to accumulate caesium isotopes is constant and species-specific (16). A faster decline in the average radioactivity of fungus of one species in the environment of one place than in the environment of another is due to the spatially variable migration of ^137^Cs between individual elements of the environment and the large or small part primarily played by local wild animals in the food chain (2).

It was assumed in the literature (31) that after radioactive fallout by which radionuclides were deposited on the surface of plants and soil, these radionuclides gradually penetrated into the lower layers, and that ^137^Cs was transported in the soil from its lower layers towards the litter and onwards through the root systems of fungi and vegetation which took it up. This is also confirmed by our research. Higher concentrations of ^137^Cs were recorded in the soil layer from 0 to 10 cm deep than in the layer from 10 to 20 cm. Soil emerges as having an ability to absorb, store and release radionuclides which depends on the physicochemical processes occurring in it.

The characterisation of the ^137^Cs transport profile made it possible to determine the places of the radionuclide’s greatest accumulation, and the tests carried out on the samples made it possible to divide the profile into four areas. The first designated area is the soil layer from 10 to 20 cm, where the lowest concentrations were measured in the entire profile. The second designated area is the soil layer from 0 to 10 cm. In this area, there was a larger reservoir of ^137^Cs, and therefore a higher concentration, although not to an extent comparable to that in the areas above it. This state of low ^137^Cs activity in the first two areas of the profile may have been caused by many years of uptake of the radionuclide by plant root systems and various formations of fungal thalli. Moreover, soil systems constitute a fairly permanent and stable structure with slow erosion, especially in forest areas (17), and therefore the movement and mixing which would facilitate the vertical transport of ^137^Cs takes place slowly. The highest concentrations were recorded in the third area of the profile, *i.e*. in the stipes of mushroom fruiting bodies. The research shows that in the last examined area, which was the mushroom caps, there was less activity than in the stipes. The fruiting bodies of mushrooms, *i.e*. the stipe and cap, are dynamically developing systems. During their growth, various nutritional elements are intensively taken from the substrate and supplied *via* very extensive mycelia (35). This explains the large increase in the concentration of ^137^Cs in the mushrooms compared to the tested soil layers. To better document the created transport profile, the transfer factor was determined; it indicated that this transfer takes place from the soil areas towards the fruiting bodies of the fungi. The obtained results, supported by literature data, attribute high variability to the TF, which results from a number of physicochemical factors occurring during the transfer of isotopes from the soil to various parts of plants and fungi.

## Conclusion

The obtained results of studies on radioactive contamination of mushrooms and the determination of ^137^Cs transport allow a broader understanding of the migration of this radiotoxic isotope in the environment. The results indicate a low health risk for consumers associated with the consumption of forest mushrooms. The limitations of these studies may be the narrow area of sampling and limited comparison of research results with those from other forest areas, the lack of seasonality of sampling over several years and the small number of samples tested.

These studies are of practical importance. The knowledge obtained from the research carried out may be used in the future to take appropriate measures to reduce the effects of repeated uncontrolled contamination with radioactive substances. Research has shown that mushrooms can be used as a tool for decontaminating radioactively contaminated areas.
